# Empagliflozin-Induced Pancreatitis: A Case Report Pattern

**DOI:** 10.7759/cureus.25189

**Published:** 2022-05-21

**Authors:** Parker Foster, Pinky Jha, Sarbagya Pandit

**Affiliations:** 1 Internal Medicine, Medical College of Wisconsin, Milwaukee, USA

**Keywords:** case-based review, adverse drug effect, acute pancreatitis, sodium-glucose cotransporter-2 (sglt2) inhibitors, empagliflozin

## Abstract

Sodium-glucose cotransporter-2 inhibitors have become a great insulin-independent approach for diabetic management. These agents have increasingly been reported to be associated with the onset of acute pancreatitis. Here, we present a suspected case of empagliflozin-induced pancreatitis. Finally, we compile previous reports of suspected cases.

## Introduction

Acute pancreatitis is characterized by a recent inflammatory reaction within pancreatic tissue. Numerous etiologies have been identified, with gallstones and chronic alcohol consumption representing the two most common [1]. Additional well-documented triggers include trauma, infection, autoimmune disease, scorpion stings, hypertriglyceridemia (>1000mg/dL), endoscopic retrograde cholangiopancreatography (ERCP), and medications. Although drug-induced pancreatitis is a well-accepted precipitant, identifying it, and further, proving an association, can be a difficult and tedious feat.

Recently developed sodium-glucose cotransporter-2 (SGLT2) inhibitors have become a great insulin-independent approach for type 2 diabetic management. The SGLT2 inhibitors currently approved by the Food and Drug Administration (FDA) include canagliflozin, dapagliflozin, and empagliflozin. This family of medications acts to reduce blood glucose levels by blocking glucose reabsorption in the proximal convoluted tubules, thereby enhancing renal excretion. Most notably, it has been reported these drugs provide an added benefit of reducing cardiovascular mortality [2,3]. The most widely reported side effects of the SGLT2 inhibitors include genitourinary tract infections, attributed to the purposeful glucosuria, as well as hypotension, due to the associated osmotic diuresis. Empagliflozin, however, has recently been increasingly implicated in the precipitation of acute pancreatitis [4-9]. Here, we report a suspected case of empagliflozin-induced pancreatitis. Finally, we conclude by reviewing reports of pancreatitis following empagliflozin initiation.

## Case presentation

A 73-year-old Caucasian female with a history of hypertension, hyperlipidemia, type 2 diabetes mellitus, and osteoarthritis presented a few hours after sudden onset, sharp left upper quadrant abdominal pain associated with multiple episodes of nausea and vomiting. The patient denied any previous event like presentation. Further workup revealed no history of pancreatitis, trauma, or alcohol abuse. Medications at time of admission included empagliflozin (substituted for dapagliflozin two months earlier), aspirin, glimepiride, metoprolol, valsartan/hydrochlorothiazide, and rosuvastatin.

**Figure 1:**
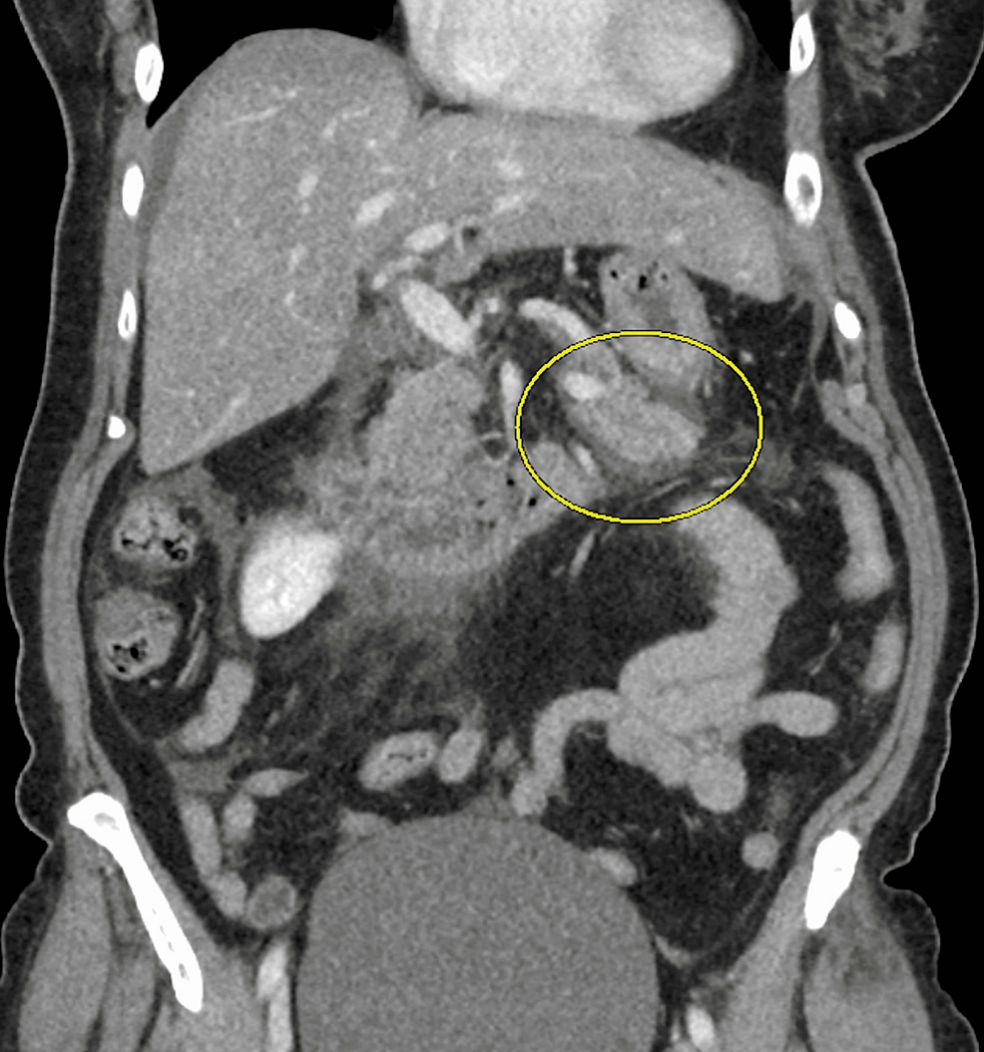
Admission CT suspects pancreatitis. The image is showing fat stranding on the tail of the pancreas (circled).

On admission, the patient was febrile to 38.3°C (101°F) with physical examination significant for diffuse abdominal tenderness. Labs were remarkable for lipase >10,000 U/L (normal: 13-60 U/L), leukocytosis to 19 K/uL, and alkaline phosphatase of 118 U/L (normal: 35-104 U/L). Triglycerides and hepatic function panel were unremarkable. Diabetes was well-controlled, with recent hemoglobin A1C of 7.2%. Abdominal CT was concerning for ileus and severe pancreatitis (Figure 1). Gallbladder and bile ducts were unremarkable on magnetic resonance cholangiopancreatography (MRCP) and abdominal CT. The patient was treated in the traditional manner, including bowel rest, intravenous fluids, and pain management. Nasogastric (NG) suction was used for ileus decompression.

**Figure 2:**
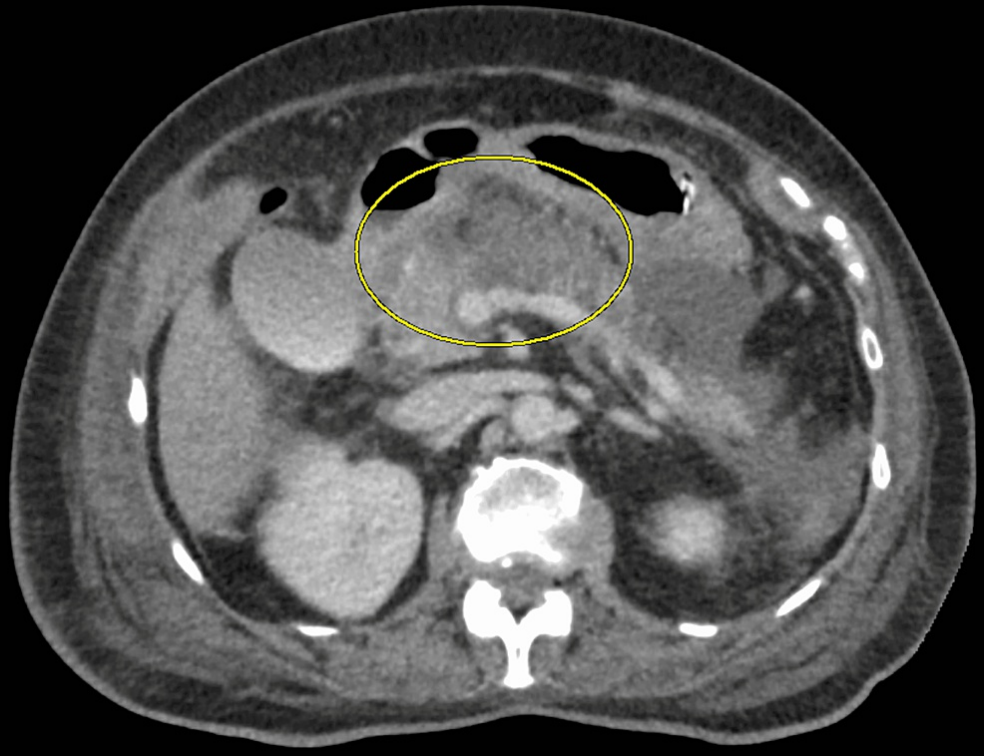
Repeat CT suspects worsening pancreatitis with possible necrosis (circled).

The patient failed to improve after five days, showing intermittent fevers, rising leukocytosis, no resolution of initial symptoms, and repeat CT positive for worsening pancreatitis (Figure 2). Repeat hepatic function panel was unremarkable. Broad spectrum antibiotics were initiated for five days for severe necrotic pancreatitis; blood cultures were later determined to be negative. The patient was started on slow tube feeds (25cc/h) via NG tube, though was unable to tolerate it. A nasojejunal (NJ) tube was subsequently placed with trickle feeds. The patient had prolonged course with slow improvement of symptoms. The patient was able to advance to clear liquid diet five days later, followed by persistent daily advancements in diet until discharge. The patient was discharged with insulin in place of empagliflozin and glimepiride, and valsartan/hydrochlorothiazide was reduced to valsartan only.

## Discussion

Acute pancreatitis was reported to be among the most common gastrointestinal causes of hospitalization in the United States [10]. Gallstones and chronic alcohol use are well-documented as the most common etiologies [1]. However, many etiologies are known to be responsible, with drug-induced causes being one of the most difficult to distinguish. To date, multiple reports have implicated the SGLT2-inhibitor empagliflozin in the onset of acute pancreatitis [4-9]. Interestingly, a large range appears to exist between empagliflozin initiation and symptom onset, with the shortest reported duration at six days, and the longest at 104 days (Table 1). Notably, our report of 60 days is in line with others. Albeit a small report size, no clear pattern seems to be present.

**Table 1:** Previous empagliflozin-induced pancreatitis reports. Duration refers to the reported length of empagliflozin use before the patient was presented. Sources that reported duration in months were converted to days, assuming one month is equal to 30 days. Age is reported in years.

Age (years)	Sex	Concurrent medications	Duration	Source
46	Male	Metformin, semaglutide	6 days	[4]
64	Female	Amlodipine, nebivolol	14 days	[5]
52	Female	Glargine, furosemide, metformin	30 days	[6]
47	Male	Atenolol, hydrochlorothiazide	60 days	[7]
51	Female	Metformin	60 days	[8]
70	Male	Atenolol, hydrochlorothiazide, ibuprofen, insulin aspart, lisinopril, ranitidine, simethicone, simvastatin	104 days	[9]

We believe this report further substantiates an association between empagliflozin initiation and the precipitation of acute pancreatitis. With no significant medical changes other than initiation of empagliflozin two months prior, our patient was found to have acute pancreatitis that could not be attributed to any other cause. Importantly, other medications that our patient was taking have been implicated in the precipitation of acute pancreatitis such as hydrochlorothiazide [11]. However, our patient had been on these medications long-term with no reported events, thus making it unlikely any of these agents were responsible. Finally, we must consider the possibility that these agents combined with SGLT-2 inhibitors have the potential to create an additive or synergistic effect toward precipitating acute pancreatitis. Large trials would be required to clearly elucidate such conclusions. However, prior reports have noted the association between empagliflozin and acute pancreatitis in the absence of medications that may react with empagliflozin, making this mechanism less likely (Table 1). Although we did not initiate a drug challenge test to confirm, we believe this episode of acute pancreatitis is most likely attributable to empagliflozin based on the clinical presentation, prior reports, and timeline [4-9].

## Conclusions

With a growing body of case reports, it is imperative physicians and related healthcare providers remain vigilant for adverse drug reactions in the setting of empagliflozin initiation. Care should be put into adequate patient education so that patients can be active participants in symptom surveillance. A lower threshold for empagliflozin discontinuation may be prudent.
